# Long-Term Frozen Storage of Urine Samples: A Trouble to Get PCR Results in *Schistosoma* spp. DNA Detection?

**DOI:** 10.1371/journal.pone.0061703

**Published:** 2013-04-16

**Authors:** Pedro Fernández-Soto, Virginia Velasco Tirado, Cristina Carranza Rodríguez, José Luis Pérez-Arellano, Antonio Muro

**Affiliations:** 1 IBSAL-CIETUS (Instituto de Investigación Biomédica de Salamanca-Centro de Investigación de Enfermedades Tropicales de la Universidad de Salamanca), Facultad de Farmacia, Universidad de Salamanca, Salamanca, Spain; 2 Departamento de Ciencias Médicas y Quirúrgicas, Facultad de Ciencias de la Salud, Universidad de Las Palmas de Gran Canaria, Las Palmas de Gran Canaria, Spain; The George Washington University Medical Center, United States of America

## Abstract

**Background:**

Human schistosomiasis remains a serious worldwide public health problem. At present, a sensitive and specific assay for routine diagnosis of schistosome infection is not yet available. The potential for detecting schistosome-derived DNA by PCR-based methods in human clinical samples is currently being investigated as a diagnostic tool with potential application in routine schistosomiasis diagnosis. Collection of diagnostic samples such as stool or blood is usually difficult in some populations. However, urine is a biological sample that can be collected in a non-invasive method, easy to get from people of all ages and easy in management, but as a sample for PCR diagnosis is still not widely used. This could be due to the high variability in the reported efficiency of detection as a result of the high variation in urine samples’ storage or conditions for handling and DNA preservation and extraction methods.

**Methodology/Principal Findings:**

We evaluate different commercial DNA extraction methods from a series of long-term frozen storage human urine samples from patients with parasitological confirmed schistosomiasis in order to assess the PCR effectiveness for *Schistosoma* spp. detection. Patientś urine samples were frozen for 18 months up to 7 years until use. Results were compared with those obtained in PCR assays using fresh healthy human urine artificially contaminated with *Schistosoma mansoni* DNA and urine samples from mice experimentally infected with *S. mansoni* cercariae stored frozen for at least 12 months before use. PCR results in fresh human artificial urine samples using different DNA based extraction methods were much more effective than those obtained when long-term frozen human urine samples were used as the source of DNA template.

**Conclusions/Significance:**

Long-term frozen human urine samples are probably not a good source for DNA extraction for use as a template in PCR detection of *Schistosoma* spp., regardless of the DNA method of extraction used.

## Introduction

Human schistosomiasis -caused by different species of digenetic trematode worms of the genus *Schistosoma*- is a severe debilitating parasitic disease that remains as a major public health problem in developing countries in tropical and subtropical areas, especially in Sub-Saharan Africa. The disease is endemic in 74 countries infecting more than 200 million people worldwide, with 732 million people at risk of infection in known transmission areas [1].

Schistosomiasis also represents an increasing problem in non-endemic areas due to the growing number of international travelers to endemic areas, expatriates and immigrants from endemic countries [2] and international cooperation programs. Besides clinical and epidemiological data the diagnosis of the disease is routinely based on microscopic demonstration of parasite eggs in patients stool or urine. This approach allows the identification of the different species of schistosomes -visualising their characteristic morphology- and is relatively inexpensive and easy to perform providing basic information on prevalence and infection intensity. However, a well known limitation of these parasitological methods is their lack of sensitivity, especially when the intensity of the infection is low, as occurs in areas of low prevalence or in individuals with recent infections [3]. Moreover, detection of parasite eggs cannot be carried out in the acute phase of schistosomiasis because production and elimination of eggs begins at two months of infection. To overcome these limitations with microscopic diagnostic, immunological methods to determine both circulating antigen or antibody levels are usually applied to patients with schistosomiasis clinical signs when parasites cannot be directly detected. Although these methods present greater sensitivity than parasitological techniques, serology-based analyses currently continue to present problems, such as obtaining schistosome antigens, inability to discriminate between active and past infection and high level of crossreactivity. Furthermore, persistence of antigens and antibodies after efficient therapy usually cause false positive results, corresponding to patients who have already eliminated the parasite [4], [5], [6].

Taking this into account, the development of new, more sensitive and specific diagnostic tools for the diagnosis of schistosomiasis are desirable and should be considered despite their higher cost and the use of special laboratory equipment [7]. In the last few years several authors have reported the successful application of polymerase chain reaction (PCR)-based methods for detection of *Schistosoma* spp. DNA in human clinical samples, such as faeces [8], [9], [10], [11], [12], sera [8], plasma [13] and urine [14].

Collection of diagnostic samples such as stools or blood is usually difficult in some population groups, but urine is a biological sample that can be collected in a non-invasive method, easy to get from people of all ages and easy to manage. As is known, urine samples contain relatively little DNA available when compared to blood samples, but currently PCR-based methods overcome this restriction and under appropriate reaction conditions yield a visible amplicon from trace amounts of DNA. Urine as a sample for PCR in diagnosis of several human infectious diseases has been successfully reported in Lyme disease [15], [16], filariasis [17], tuberculosis [18], [19], malaria [20], leishmaniasis [21] and schistosomiasis [14], although at present it is still not widely used. Possibly, one of the reasons for this could be due to the high variability in the reported efficiency of detection, as occurs for example in tuberculosis, as a result of the high variation in urine specimen storage [19]. On the other hand, in Lyme disease diagnosis certain conditions for handling, DNA preservation and extraction methods from human urine samples have been found to be critical to the performance of successful PCR assays [16]. Moreover, a recent study on stability of urinary DNA in stored urine in two populations over 28 days –measured at different temperatures and with or without the addition of a preserving solution-, showed that the stability of human DNA in urine is dependent on geographic origin [22]. Thus, there are many conflicting data in the literature, not only on how to store urine samples to preserve stability of DNA, but also in different methods for subsequent extraction of such samples in order to obtain effective results in molecular diagnosis.

In a previous work our group developed a sensitive and specific PCR-based approach for the amplification of defined regions from 28S ribosomal DNA yielding two genus-specific fragments (877 bp and 1032 bp) and a 350 bp species-specific fragment for *S. mansoni.* This PCR showed to be useful in schistosomiasis diagnosis when urine from eighteen patients infected with different species of schistosomas were used as the template source [14]. However, in that work the method of DNA extraction from urine resulted laborious, requiring large volumes of samples and, additionally, high cost.

In the present study, we evaluate different DNA extraction methods from human urine samples in order to assess the PCR effectiveness for *Schistosoma* spp. detection in a larger series of patientś urine samples after long-term frozen storage from an endemic area for schistosomiasis.

## Materials and Methods

### Ethics Statement

The study protocol was approved by the institutional research commission of the University of Salamanca. Ethical approval was obtained from the Ethics Committee of the University of Salamanca (protocol approval number 48531), which approved the animal protocol and also the informed consent procedure. Animal procedures in this study complied with the Spanish (Real Decreto RD1201/05) and the European Union (European Directive 2010/63/EU) guidelines on animal experimentation for the protection and humane use of laboratory animals and were conducted at the accredited Animal Experimentation Facility (Servicio de Experimentación Animal) of the University of Salamanca (Register number: PAE/SA/001). The human urine samples used in this study were obtained as part of public health diagnostic activities and stored at CIETUS (University of Salamanca). Samples were already collected before the start of the study and were tested as anonymous samples. Participation of healthy urine donors (laboratory staff) was voluntary. Participants were given detailed explanations about the aims, procedures and possible benefit of the study. Written informed consent was obtained from all subjects and samples were coded and treated anonymously.

### Urine Samples

#### Patientś urine samples

A total of seventy-three human urine samples with confirmed schistosomiasis through the detection of parasite eggs in stools (by Kato-Katz technique) or urine (by sedimentation or filtration methods) were collected from sub-Saharan immigrants at Hospital Universitario Insular (Las Palmas de Gran Canaria, Spain), including 55 patients infected with *S. haematobium* -counting two co-infections with *S. mansoni*- and 18 patients infected with *S. mansoni.* Untreated human urine samples were frozen at −80°C until use for a minimum of 18 months up to 7 years.

#### Mice urine samples

Nine 6-week old female BALB/c mice were used as the source for urine samples. Six mice were each infected subcutaneously with 150 *S. mansoni* cercariae [23] and urine samples were taken and pooled at week 3, 6 and 9 post-infection (p.i.), respectively. In order to confirm infections, mice were sacrificed at week 9 p.i. and worms were recovered in each infected mouse after conventional portal-hepatic perfusion. Three uninfected mice were used as the control group. All mice urine samples were stored at −80°C for at least twelve months before use.

#### Artificial urine samples

Fresh urine was taken from healthy staff donors, divided into aliquots and then artificially contaminated with different amounts of adult *S. mansoni* DNA. Three sets of samples were prepared. Set 1 consisted in aliquots of 250 µL, 500 µL and 1 mL each of fresh urine contaminated with decreasing amounts of DNA: 64 ng, 32 ng, 16 ng, 10 ng–1 ng, 0.500 ng, 0.250 ng and 0.125 ng. Set 2 consisted in aliquots of 2 mL, 3 mL and 5 mL each of fresh urine contaminated with 1.25 ng, 2.50 ng, 5 ng, 10 ng, 20 ng and 40 ng of DNA. Set 3 consisted in aliquots of 25 µL each of fresh urine contaminated with 1.25 ng, 2.50 ng, 5 ng and 10 ng of *S. mansoni* DNA. All artificial urine samples were prepared when required and directly processed for DNA extraction without carrying out any prior storage process.

### DNA Extraction

#### Schistosoma mansoni DNA extraction

*S. mansoni* DNA was extracted from frozen adult worms using DNeasy® Blood & Tissue Kit (Qiagen®, Hilden, Germany) following the manufactureŕs instructions. DNA samples were diluted at 1 µg/µL in ultrapure water and stored at −20°C until use. DNA obtained was used as a positive control in all PCR assays as well as in the preparation of artificial urine samples as mentioned above.

### Urine Samples DNA Extraction

Two techniques were tested for DNA extraction from urine samples followed by PCR amplification: a method by using two Chelex-100® chelating resin based extraction protocols and three commercial available DNA extraction kits.

For the single-tube Chelex-100® based DNA extraction method we used two procedures both consisting in boiling whole or formerly centrifuged artificial urine samples in a variable concentration suspension of autoclaved PCR-grade water and Chelex-100® resin (Bio-Rad Laboratories, CA, USA). Each procedure was carried out as follows.

In the first procedure whole artificial urine aliquots of 250 µL, 500 µL and 1 mL (set 1) were mixed and vortexed with an equal volume of Chelex-100® resin at 5% or 20% (w/v), respectively. Then, the mixtures were heated for 10 min at 100°C and centrifuged at 13000 rpm for 4–5 min to pellet out the Chelex-100® resin and retain the supernatant for PCR.

In the second procedure artificial urine aliquots of 250 µL, 500 µL and 1 mL (set 1) were previously centrifuged at 10000 rpm for 5 min and the supernatant was discarded. Then, an equal volume of Chelex-100® resin at 5% or 20% (w/v) was added to the urine sediment formed, vortexed vigorously and the mixtures were heated for 10 min at 100°C. After this, the boiled mixtures were centrifuged at 13000 rpm for 4–5 min to pellet out the Chelex-100® resin and the supernatant was used in PCR assays. Same protocol was also assayed with three additional artificial urine aliquots of 500 µL each using 100 µL of Chelex-100® resin at 5%, 30% or 40% (w/v), respectively.

These procedures were also applied for DNA extraction from whole urine samples randomly collected from five patients infected with *S. mansoni* and with formerly centrifuged urine samples from mice. For human urine samples, a volume of 100 µL, 250 µL and 500 µL of Chelex-100® resin at 5% or 20% (w/v) was added to aliquots of 250 µL and 500 µL each, respectively. For mice urine samples a volume of 100 µL Chelex-100® resin at 5% (w/v) was added to aliquots of 150 µL or 500 µL pooled at 3 week p.i. and 250 µL or 500 µL pooled at 6–9 weeks p.i.

In addition, as the Chelex-100® based DNA extraction method is unable to remove possible PCR inhibitors (i.e. proteins, haemoglobin), we also attempted to treat each of the five urine samples from patients infected with *S. mansoni* with proteinase K in order to degrade as much of the proteins potentially present in the samples which could be detrimental to downstream processes. For this, a volume of 250 µL each of the patientś urine samples were also treated with 20 µL of 20 mg/mL proteinase K for 2 h at 56°C in gentle vortexing, centrifuged at 13000 rpm for 5 min and a volume of 100 µL Chelex-100® resin at 5% (w/v) was added separately to the supernatant and the pellet obtained. DNA obtained from all urine samples assayed using the Chelex-100® based DNA extraction method was used as template and tested by species-specific PCR SmF-SmR.

On the other hand, three commercial available DNA extraction kits were tested firstly for their ability to recover DNA from various dilutions of artificial urine samples: NucleoSpin® DNA Trace Kit, FitAmp™ Urine DNA Isolation Kit and Urine DNA® Isolation Kit. Comparison of costs, volumes of samples and eluted DNA, processing time and other miscellaneous aspects among the evaluated DNA extractions kits are shown in Table 1. To evaluate the kits artificial urine samples prepared as mentioned above in set 2 were used. For each kit evaluated the manufacturerś protocol and recommended modifications to increase DNA recovery from urine samples were followed and employed as noted below. In the three kits the DNA extraction is based on spin capture column chromatography. Briefly, the sample was added to the column (lysed prior with a lysis reagent and/or proteinase K) to allow DNA to bind, several wash steps were used to remove inhibiting substances and then the DNA was eluted from the column. The NucleoSpin® DNA Trace kit is not specifically designed for urine samples but is designed for the preparation of genomic DNA from small amounts of any tissue, cells and forensic samples. The NucleoSpin® DNA Trace F columns (F means funnel) included in the kit are designed for collecting small amounts of nucleic acids from large volumes because these columns are shaped like a funnel combining a large volume capacity with a small diameter of the binding membrane. The FitAmp™ Urine DNA Isolation Kit and the Urine DNA® Isolation Kit are specifically designed for urine samples. Instructions for the Urine DNA® Isolation Kit recommended eluting the column twice with the same elution buffer (75 µL) to increase DNA yield. Additionally, the volume of urine sample used with this kit could be reduced to 25 µL, as indicated by the manufacturer. To verify, we attempted DNA extraction with the Urine DNA® Isolation Kit from as little as 25 µL of fresh artificial urine samples contaminated with 1.25, 2.5, 5 and 10 ng of *S. mansoni* DNA (set 3) as well as from experimentally infected mice.

**Table 1:** Comparison of cost, processing time and other miscellaneous aspects among the evaluated DNA extraction methods.

	Chelex-100*®* MolecularGrade Resin	NucleoSpin*®* DNATrace Kit	FitAmp™ Urine DNAIsolation Kit	Urine DNA*®*Isolation Kit
Manufacturer	BIO-RAD	MACHEREY-NAGEL	EPIGENTEK	NORGEN BIOTEK CORPORATION
Cost	200 € (100 g)	478 € (25 samples)	210 € (50 samples)	325 € (50 samples)
			388 € (100 samples)	
Sample vol.	variable	4–8 mL	5 mL	25 µL–1.75 mL
Recovered extraction vol.	variable	100 µL	8–18 µL	75 µL+75 µL
Processing time^a^	15–20 min	60 min+ incubation (≥1 h)	60 min	90 min
Room temperature duration	>1 year	1 year	6 months	1 year

a Time required to obtain extracted DNA, including hands-on time and incubation periods.

Moreover, for a first extraction trial in frozen patientś urine samples using the Urine DNA® Isolation Kit, eight of our parasitological positive human urine samples were randomly selected, including four *S. mansoni* and four *S. haematobium* positive samples. For *S. mansoni* and *S. haematobium* infected samples, two aliquots of 1.75 mL - as recommended by the manufacturer- as well as 50 µL –lesser than recommended- each were used separately for DNA extraction and specific PCRs were performed. Finally, the Urine DNA® Isolation Kit was further used for DNA extraction using aliquots of 1.75 mL from all 73 frozen patientś urine samples included in the study. DNA obtained in this way was used as template and tested twice by genus-specific PCR CF2-CR2 and species-specific PCR SmF-SmR.

### DNA Quantification

After using the DNA extraction methods mentioned, total DNA concentrations from *S. mansoni* adult worms as well as from all urine samples assayed were determined using a Nanodrop ND-1000 spectrophotometer (Nanodrop Technologies). Each sample (2 µL) was measured twice and the DNA concentrations averaged. In order to look for protein contaminations a common purity check by measuring the A_260_/A_280_ ratio was made.

### Polymerase Chain Reaction

Two protocols were followed with some modifications. The first PCR was carried out according to the protocol detailed by Sandoval *et al.* [24]. Briefly, two primer pairs were used in order to amplify *Schistosoma* spp. 28S rDNA: CF1/CR2 and CF2/CR2, giving genus-specific PCR products of 877 and 1032 base pairs (bp), respectively. We also used the specific primers for the amplification of the 28S rDNA region from *S. mansoni* (SmF/SmR) giving a species-specific PCR product of 350 bp. Routinely, PCR reactions were performed in a final volume of 20 µL, containing 2 µL of 10X reaction buffer, 3 mM MgCl_2_, 2.5 U of Taq polymerase (Bioron, GmbH, Germany), 2 µM of each primer (TIB-MOLBIOL, Germany), 0.5 mM dNTPs (Eppendorf) and 1 µL of template DNA. PCR cycling parameters consisted of 3 min at 94°C followed by 35 cycles of 30 s at 94°C, 20 s at 65°C, and 20 s at 72°C with a final extension at 72°C for 7 min. Using the primers mentioned above, several modifications were tested in PCR assays to improve results with human urine samples studied, such as increasing the amount of template DNA (5 µL *vs.* 1 µL) or trying other cycling parameters by a touchdown PCR (TD-PCR) performed as follows: 94°C for 1 min, a touchdown program for 18 cycles (successive annealing temperature decrements of 1.0°C every 2 cycles, consisting in 94°C for 20 s, 70°C-62°C or 65°C-57°C for 20 s, 72°C for 30 s), 15 similar cycles (except for the annealing temperature at 57°C) and a final extension at 72°C for 10 min. The second PCR protocol, described by Kato-Hayashi *et al.* [25], was used for the amplification of different regions of the cytochrome c oxydase subunit (*cox1*) gene of *Schistosoma* spp., using common primer pairs CF/CR for *Schistosoma* spp. (254 bp) and specific primer pairs SmF/CR and Sh/CR for *S. mansoni* (479 bp) and *S. haematobium* (365 bp), respectively. Briefly, PCR was carried out in a final volume of 20 µL containing 2 µL of 10X reaction buffer, 1.5 mM of MgCl_2_, 0.2 mM of each dNTP (Eppendorf), 0.4 U of Taq DNA polymerase (Bioron, GmbH, Germany), 0.5 µM of each primer (TIB-MOLBIOL, Germany) and 1 µL of template DNA. The PCR reactions were performed at 94°C for 2 min, followed by 35 cycles of 30 s at 94°C, 30 s at 58°C, 60 s at 72°C and a final cycle at 72°C for 7 min. Following the authorś recommendations for DNA amplification in biological samples (sera and/or urine), several modifications were performed in order to improve results with the studied samples, such as varying concentrations of MgCl_2_ (2.0, 2.5 mM) and primers (1.0, 1.5 and 2.0 µM), units of Taq polymerase added (0.5, 0.75 and 1.0 U) and increasing the number of reactions up to 50 cycles. In all PCR assays, a positive (DNA of *S. mansoni*) and a negative (ultrapure water or non-contaminated urine) controls were included. The amplified products were visualized by electrophoresis on ethidium bromide-staining 1.2% agarose gels and then recorded by digital photography.

## Results

### PCR Results in Urine Samples Using Chelex-100® Based DNA Extraction Method

In general, the results obtained in PCR amplification of *S. mansoni* species-specific product of 350 bp using both whole and formerly centrifuged artificial fresh urine samples using Chelex-100® based DNA extraction protocols were rather irregular and repetitive. In PCR tests using whole urine samples only positive results were obtained by using a starting volume of urine of 500 µL and performing DNA extraction with an equal volume of Chelex-100® resin at 5% or 20%.

In PCR tests for formerly centrifuged urine several positive results were obtained by using starting volumes of urine of 250 µL, 500 µL and 1 mL and performing DNA extraction with 100 µL of Chelex-100® resin at 5% (for the starting volumes indicated) and 20% (only for a starting volume of urine of 500 µL). Specific products of 350 pb were obtained in aliquots contaminated with decreasing amounts (64 ng to 0.125 ng) of *S. mansoni* DNA (set 1) using a starting volume of formerly centrifuged urine of 500 µL and performing DNA extraction with 100 µL of Chelex-100® resin at 5%. However, positive results were not reproducible when PCR Smf-SmR was attempted repeatedly. No amplifications were obtained using the resin at concentrations above 20% (30% and 40%).

No positive PCR results using *S. mansoni* species-specific primers (350 bp) were obtained when whole urine samples from five patients infected with *S. mansoni* and formerly centrifuged urine samples from mice experimentally infected with the parasite were tested. PCR SmF-SmR also failed to produce amplicons of the expected size in whole and formerly centrifuged patientś urine pretreated with proteinase K before both Chelex-100® resin at 5% DNA extraction protocols were attempted.

### PCR Results in Urine Samples Using the Commercial DNA Extraction Kits

**Figure 1:**
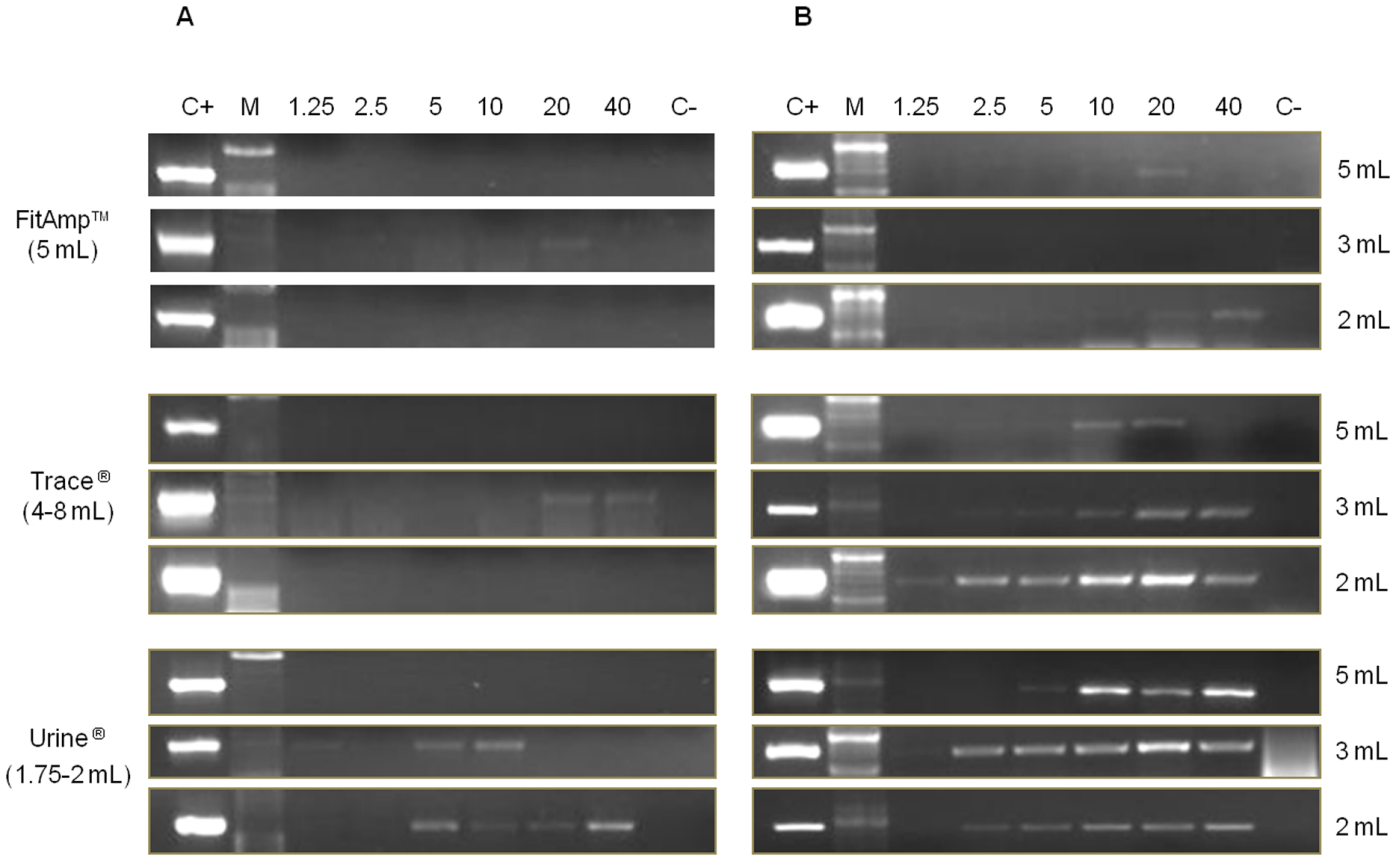
PCR for *Schistosoma* spp. *and S. mansoni* in artificial urine samples as DNA source using commercial kits as DNA extraction methods. Agarose gel electrophoresis of *Schistosoma* genus-specific PCR products of 877 bp (A) and *S. mansoni* species-specific products of 350 bp (B), respectively, obtained in fresh healthy artificial urine samples using three commercially available DNA extraction kits: FitAmp™ Urine DNA Isolation Kit (FitAmp), NucleoSpin® DNA Trace Kit (Trace), and Urine DNA® Isolation Kit (Urine). 1.25, 2.5, 5, 10, 20, 40: ng of DNA added to aliquots of 5 mL, 3 mL and 2 mL of urine. Volumes recommended by the manufacturers to increase DNA recovery from urine samples are indicated in brackets; C+, positive control (*S. mansoni* DNA, 1 ng/µL); C−, negative control (ultrapure water); M, molecular weight marker XIII (Roche).

Comparative PCR results with *Schistosoma* genus-specific (877 bp) and *S. mansoni* species-specific (350 bp) primers obtained in fresh artificial urine samples (set 2) after using the commercial DNA extraction kits are shown in Figure 1. Despite manufacturers recommendation to use a volume of 5 mL of urine with the Urine DNA Isolation FitAmp™ Kit and 4–8 mL with the DNA Trace NucleoSpin® Kit for maximum efficiency in DNA extraction (Table 1), the use of the genus-specific primer pairs CF1-CR2 failed to produce amplicons with both the two kits when they were used with aliquots of 5 mL of urine. As could be expected, using the Urine DNA Isolation Kit® with 5 mL of urine (a higher volume than 1.75–2 mL recommended by the manufacturer) no amplification was obtained by PCR CF1-CR2. A few amplicons of the expected size (877 bp) were obtained when aliquots of 3 mL were used with the three kits for DNA extraction. PCR CF1-CR2 produced amplicons when using a volume of 2 mL only with the Urine DNA Isolation Kit®.

The use of the species-specific primer pairs SmF-SmR only failed to produce amplicons of the expected size when aliquots of 3 mL of urine were used for DNA extraction using the FitAmp™ Urine DNA Isolation Kit. In general, the most successful kit in extraction of detectable DNA by genus and specific PCRs was the Urine DNA® Isolation Kit using a similar volume to that recommended by the manufacturer as optimal for DNA extraction (1.75 mL–2 mL).

**Figure 2:**
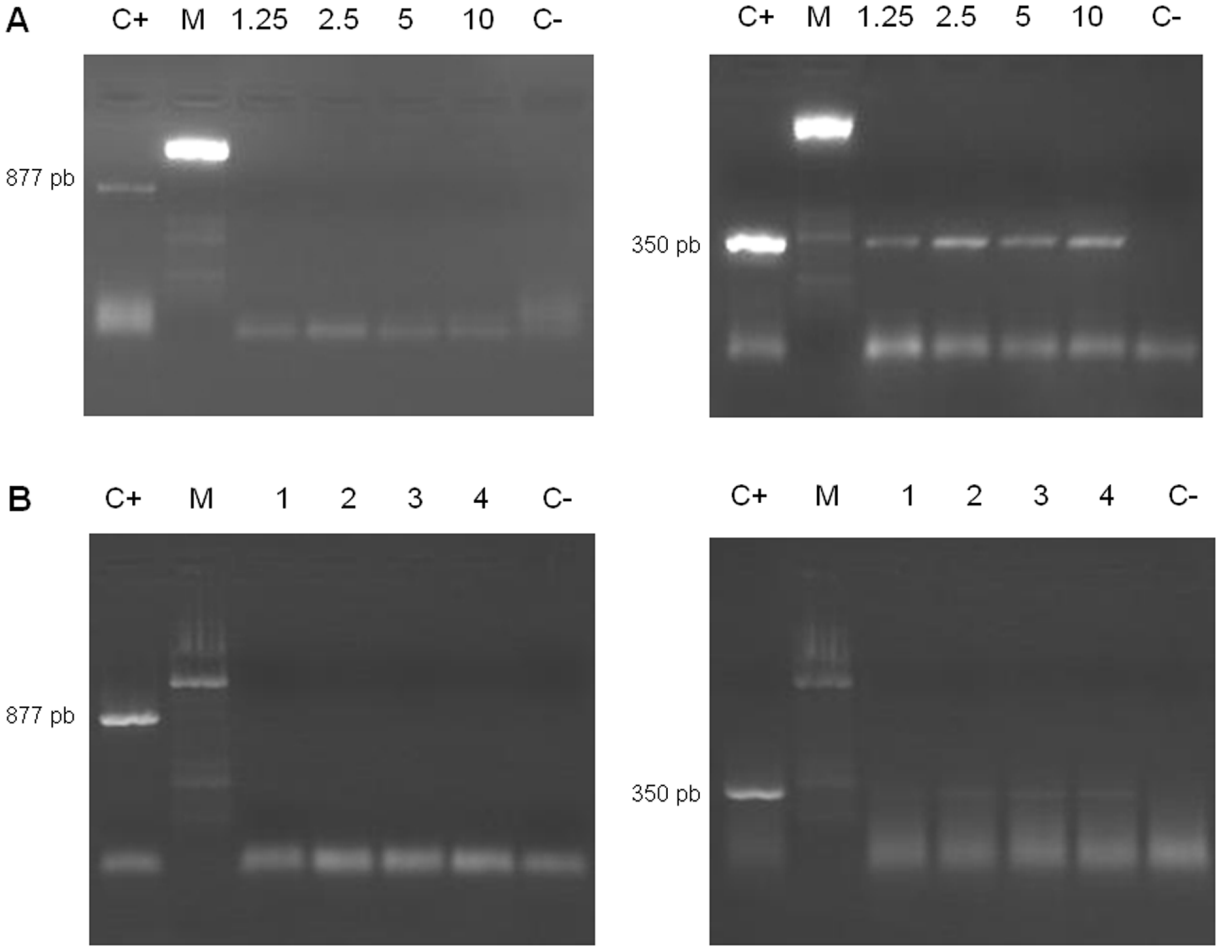
PCR for *Schistosoma* spp. *and S. mansoni* in aliquots of 25 µL of artificial urine samples and mice urine samples as DNA source using the Urine DNA® Isolation Kit as the DNA extraction method. Agarose gel electrophoresis of *Schistosoma* genus-specific PCR products of 877 bp and *S. mansoni* species-specific products of 350 bp in fresh healthy artificial urine samples (A) and in urine from mice (B) using aliquots of 25 µL for DNA extraction. 1.25, 2.5, 5 and 10: ng of DNA added to aliquots; 1–4: numbers of mice; C+, positive control (*S. mansoni* DNA, 1 ng/µL); C−, negative control (ultrapure water); M, molecular weight marker XIII (Roche).

When the specific PCRs (CF1-CR2 and SmF-SmR) were applied on DNA extracted using the Urine DNA Isolation Kit® from as little as 25 µL of fresh artificial urine samples as well as from experimentally infected mice, only the second primer pair yielded PCR products of the expected size. Amplicons of 350 bp obtained from fresh artificial urine samples were much brighter and more concentrated than those obtained from frozen urine of mice (Figure 2).

**Figure 3:**
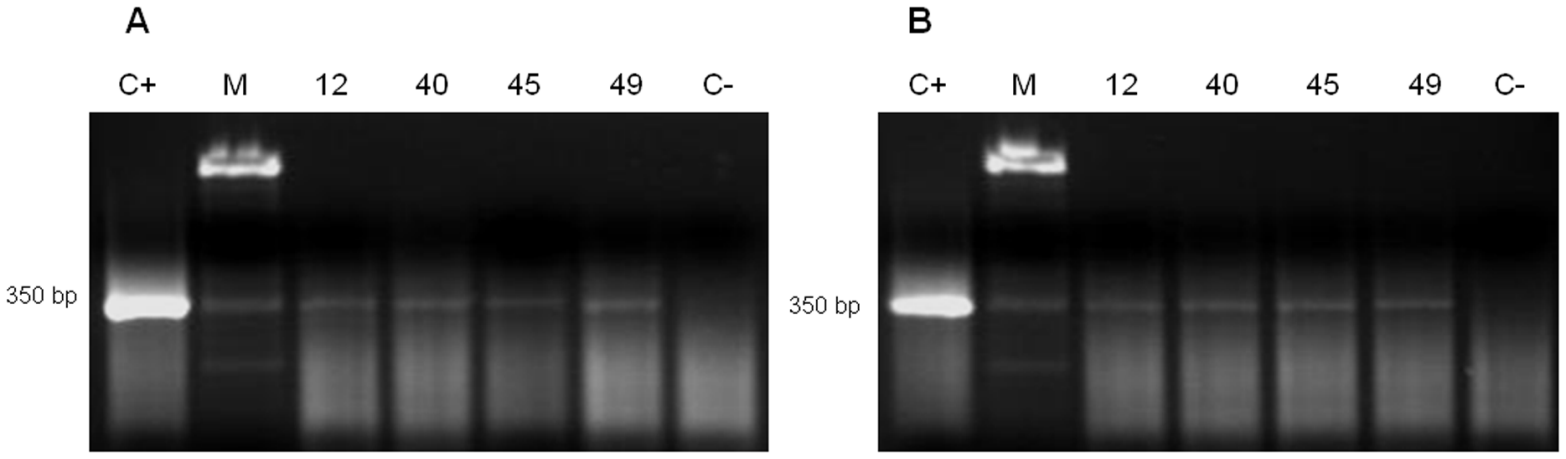
PCR for *S. mansoni* in patientś urine samples infected with *S. mansoni* as DNA source using the Urine DNA® Isolation Kit as the DNA extraction method. Agarose gel electrophoresis of *Schistosoma* species-specific PCR product of 350 bp obtained in four human urine samples from patients infected with *S. mansoni* (12, 40, 45, 49) using a volume of 1.75 mL (A) and 50 µL (B) of urine for DNA extraction using the Urine DNA*®* Isolation Kit; C+, positive control (*S. mansoni* DNA, 1 ng/µL); C−, negative control (ultrapure water); M, molecular weight marker XIII (Roche).

**Figure 4:**
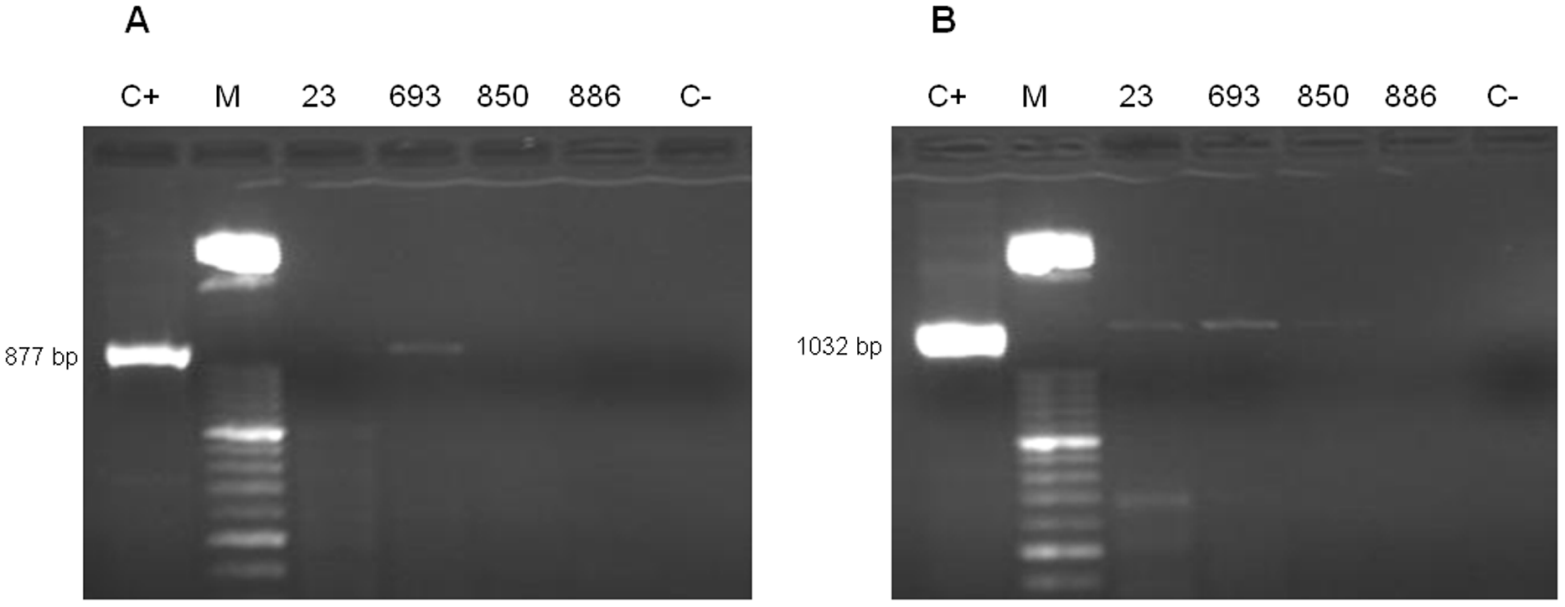
PCR for *Schistosoma* spp. in patientś urine samples infected with *S. haematobium* as DNA source using the Urine DNA® Isolation Kit as the DNA extraction method. Agarose gel electrophoresis of *Schistosoma* genus-specific PCR CF1-CR2 (A) and CF2-CR2 (B) products obtained in four human urine samples of patients infected with *S. haematobium* (numbers 123, 693, 850, 886) using a volume of 1.75 mL of urine for DNA extraction using the Urine DNA*®* Isolation Kit; C+, positive control (*S. mansoni* DNA, 1 ng/µL); C−, negative control (ultrapure water); M, molecular weight marker XIII (Roche).

The results for a first extraction trial using the Urine DNA Isolation Kit® from aliquots of 1.75 mL and 50 µL in randomly selected frozen human urine samples, including four *S. mansoni* and four *S. haematobium,* are presented in Figures 3 and 4, respectively. For *S. mansoni* infected samples, only PCR SmF-SmR, but not PCR CF1-CR2, was positive when both volumes were used as the DNA source for extraction (Figure 3). For *S. haematobium*, PCR positive results were obtained in 2/4 and 3/4 *S. haematobium* infected samples when genus-specific PCRs CF1-CR2 (877 bp) and CF2-CR2 (1032 bp), respectively, were performed using aliquots of 1.75 mL (Figure 4). In this case no PCR results were obtained when aliquots of 50 µL were used as the DNA source for extraction.

The results obtained when tested twice all 73 patientś urine samples by genus-specific PCR CF2-CR2 and species-specific PCR SmF-SmR after using the Urine DNA® Isolation Kit as DNA extraction method are presented in Table 2. No more PCR positive results were obtained when higher amounts of DNA template were added to reactions or when different PCR conditions, such as touch-down PCR, were applied. However, it should be noted that in all these PCR trials, positive controls (*S. mansoni* DNA; 1 ng/µL) were always successfully amplified (data not shown). When attempted PCR described by Kato-Hayashi *et al.* [25] for testing patientś urine samples, this PCR failed to produce positive results, even following the authorś recommendations for DNA amplification in urine samples as mentioned above.

**Table 2:** Specific PCR results in human urine samples collected from 73 immigrants with parasitologically confirmed schistosomiasis.

PCS^a^	No. of samples	PCR CF2-CR2		PCR SmF-SmR			
		1^st^ assay	2^nd^ assay	1^st^ assay	2^nd^ assay	Positive PCR CF2-CR2/PCS	Positive PCR SmF-SmR/PCS
*S. mansoni*	18	0	1 (co-infection)	1	2	1/18	3/18
*S. haematobium*	55	8	1 (co-infection)	0	0	9/55	0/55
Total	73	8	2	1	2	10/73	3/73

a Parasitologically Confirmed Schistosomiasis.

## Discussion

Schistosomiasis remains a serious worldwide public health problem and, at present, a sensitive and specific assay for routine diagnosis of schistosome infection is not yet available. Methods that allow early diagnosis, both in acute and chronic stages, are a prerequisite for effective disease control. It is well known that the current gold standard method for diagnosis is the microscopic counts of parasite eggs excreted in faeces or urine, but this parasitological method is unable to detect prepatent infection or low levels of infection particularly found in children, people with chronic infection where eggs are trapped in tissues rather than excreted and in areas of low schistosome levels [26], [27], [28]. On the other hand, many diagnostic methods for schistosomiasis based on the specific detection of antigens or antibodies have been developed, but lack of sensitivity and specificity remain a problem and a considerable number of schistosomiasis patients can be incorrectly diagnosed [29]. Moreover, false positives, mainly due to cross-reactivity of the currently used serological tools, are also very common [30].

In this context, the potential for detecting schistosome-derived DNA by PCR-based methods in human clinical samples is currently being investigated as a more sensitive and specific diagnostic tool with potential application in the routine schistosomiasis diagnosis. Among human clinical samples which can possibly be obtained of DNA for diagnostic purposes, there are a number of advantages to using the DNA found in urine, including non-invasive sample collection, easy to get from people of all ages and easy in management. It has been demonstrated that small amounts of cell-free circulating DNA are able to pass the kidney barrier and end up in urine [31], [32], [33]; furthermore, this circulating DNA from the bloodstream that passes into the urine can be isolated and used in diagnostic applications. However, the current extraction methods for isolating DNA from urine have some drawbacks, such as being time consuming and tedious, a great volume of sample is required and result in trace amounts of DNA extracted [34]. Further, if a specific commercially available kit is used for DNA extraction, the process could be very expensive to use when a large number of samples must be tested.

Taking all this into account, in this study we evaluated different DNA extraction methods for their ability to isolate DNA from small volumes of human urine samples in order to assess the PCR effectiveness for *Schistosoma* spp. detection in patients with parasitologically confirmed schistosomiasis. All the clinical urine samples included in this retrospective study had been frozen for a long time before use.

The first DNA extraction method applied was a single tube resin Chelex-100®-based method. Chelex-100® is a chelating resin which uses ion exchange to bind transition metal ions. During the extraction process the alkalinity of the solution and the act of boiling the solution breaks down the cells and allows the chelating groups to bind to the cellular components protecting the DNA from degradation [35]. We tried the Chelex-100® based DNA extraction method because it is cheap and quick, it does not require multiple tube transfers avoiding contamination and it does not use toxic organic solvents such as phenol-chloroform [36]. Furthermore, this method has been successfully reported in DNA extraction from several organisms for PCR assays [37], [38], [39]. However, when we firstly evaluated this simple method for DNA extraction from fresh artificial human urine samples the PCR results were always rather irregular and repetitive. As the Chelex-100® based DNA extraction method is unable to remove possible PCR inhibitors, the high variability and scarcity in the results obtained could be due to the presence of several inhibitors in samples than can interfere in subsequent PCR analysis. In fact, while the Chelex-100® based DNA extraction method seemed to yield enough quantity of DNA, nevertheless the A_260_/A_280_ ratio always indicated a high protein contamination (data not shown). The best quality in detectable DNA by PCR using Chelex-100® based DNA extraction method was obtained when a 100 µL suspension of 5% resin in autoclaved PCR-grade water was added and mixed thoroughly with the pellet after prior centrifugation of 500 µL urine. Perhaps, this volume of Chelex-100® resin suspension could be the most suitable for DNA extraction from a small volume of urine as 500 µL and centrifugation of urine samples as a previous step to the addition of Chelex-100® resin also could provide the removal of an important number of possible inhibitors. Lamentably, conflicting and irreproducible PCR results were obtained when we attempted DNA extraction repeatedly; as a result, the Chelex-100® based DNA extraction method was finally discarded to obtain DNA as a source for *Schistosoma* spp. detection.

A similar simple procedure for extracting *S. mansoni* DNA from artificially contaminated human urine samples has been recently reported as successful by Enk et al. [40]. In this case, authors used InstaGene matrix® (BioRad) -made with a specially formulated 6% w/v Chelex resin- after a salting-out pretreatment of urine samples with NaCl and subsequent DNA precipitation with ethanol. Detectable DNA by PCR was extracted when it was at a concentration of 1.28 pg DNA/mL, revealing the high efficiency of this procedure. Thus, using a simple method involving a chelating resin in combination with a high sensible PCR it is possible to detect *S. mansoni* in artificial urine samples as a DNA source. More recently, the same authors used this simple DNA extraction method in frozen patientś urine samples from an endemic area of Schistosomiasis with very good results [41]. In our work, despite we obtained PCR specific-*S. mansoni* products using just a simple Chelex-100® based DNA extraction method in fresh artificial human urine samples, this method failed to obtain a good quality detectable DNA for testing the samples repeteadly by PCR. Moreover, Chelex-100® based DNA extraction method also failed to produce detectable DNA by PCR in patientś urine samples frozen for a long time even after treatment with proteinase K to degrade potential proteins acting as PCR inhibitors. This could be due to the freezer-induced urinary precipitates which almost always develop after the urine samples are frozen overnight or longer [42], which could probably be acting as inhibitors of PCR reaction. Certainly, a higher quality of detectable DNA by PCR in our patientś urine samples could be obtained if a technique based on salting-out and resin procedure would be applied as successfully reported by Enk *et al* [40], [41]. Unfortunately, in that study the freezing time of urine samples is not indicated but we think that our patientś urine samples have been stored frozen much longer. In this sense, it should be desirable to investigate such technique with long-term frozen human urine samples to improve results.

Extraction methods not only have to ensure that DNA is efficiently extracted from each sample, they also have to remove inhibitors which may interfere with subsequent downstream processes. This is especially critical for urine specimens, since urine has been found to be a particularly difficult substrate for PCR [34]. Theoretically, DNA extracted using spin capture column chromatography should be the cleanest, containing the least PCR-inhibitory substances. Thus, in order to obtain a good quality detectable DNA that did not compromise PCR sensitivity for testing our clinical samples, we also evaluated three well known available commercial kits for DNA extraction that allow purifying and recovering DNA from urine specimens. Both the Urine DNA Isolation Kit® and the Urine DNA Isolation FitAmp™ Kit are specifically designed for using with urine samples. The DNA Trace NucleoSpin® Kit is designed for DNA extraction from traces of several types of biological samples. This kit is more tedious, time consuming and expensive than others but we included it in our comparative study because it worked well in extracting DNA from human urine samples for PCR detection of *Schistosoma* spp. in a previous work reported by our group [14]. To assess the ability of the commercial kits for extracting detectable DNA by PCR firstly we used fresh artificial urine samples for DNA extraction and then genus-specific PCR (CF1-CR2) and species-specific PCR (SmF-SmR) were performed. Surprisingly, the results obtained with the kits were unexpected and discordant when volumes recommended by the manufacturers to increase DNA recovery from urine samples were used. The Urine DNA Isolation FitAmp™ Kit was the most inefficient to extract detectable DNA by genus and specific PCRs even using a volume of 5 mL for DNA extraction as recommended by manufacturers. The DNA Trace NucleoSpin® Kit also failed to obtain detectable DNA by genus-specific PCR using a volume of 5 mL (in the range of suitable volume as recommended by manufacturers); unexpectedly, this kit worked well to extract detectable DNA by species-specific PCR when a lesser volume of urine samples (2 mL) than recommended (4–8 mL) was used for DNA extraction. In general, the Urine DNA Isolation Kit® showed the highest efficiency to obtain detectable DNA by genus and specific PCRs when a suitable volume as recommended by the manufacturers was used. On the other hand, the supplied protocol for the Urine DNA® Isolation Kit suggests that the use of as little as 25 µL of urine can provide enough DNA of a high quality for PCR detection. Hence, to verify this we attempted firstly for DNA extraction from 25 µL of fresh artificial urine samples as well as from 25 µL of one-year-frozen urine from mice as the DNA source and then genus and specific PCRs were applied. Using this little volume of urine as the DNA source for extraction only the 350 pb *S. mansoni* specific product was successfully amplified. The amplicons obtained from fresh artificial urine samples resulted much brighter and more concentrated than those obtained from frozen urine of mice suggesting that the quality of extracted DNA for PCR purposes decreases when urine samples have been frozen for at least one year. Being aware of this drawback we attempted the DNA extraction using the Urine DNA Isolation Kit® from eight of our long-term frozen parasitological positive human urine samples, including four *S. mansoni* and four *S. haematobium* infected samples. For this trial we used aliquots of 1.75 mL (as optimal volume recommended by manufacturer) as well as aliquots of 50 µL from each selected urine sample. For *S. mansoni* infected samples positive results were obtained by PCR SmF-SmR when both volumes were used as the DNA source for extraction. However, very faint PCR products of 350 pb with a clearly visible smear were visualized in electrophoresis suggesting once again, as observed for one-year-frozen urine from mice, that the quality of extracted DNA for PCR assays decreases when urine samples have been frozen for a long time. For *S. haematobium* infected samples also very faint PCR amplicons of 877 pb and 1032 pb were obtained when aliquots of 150 µL were used as the DNA source for extraction. Unexpectedly, no PCR results were obtained when a volume of 50 µL was used for DNA extraction.

Considering the foregoing, in order for urine DNA to be used for *Schistosoma* spp. PCR detection in clinical samples included in our study we finally decided to use the Urine DNA Isolation Kit® as the method for DNA extraction considering: *i)* the most consistently positive results at testing artificially and human urine samples, *ii)* the possibility of using small volumes of urine as the DNA source (1.75 mL, 50 µL and 25 µL), *iii)* the easy management and *iv)* cost per sample. For PCR assays, in addition to species-specific PCR SmF-SmR for *S. mansoni* detection, we used the genus-specific PCR CF2-CR2 for *Schistosoma* spp. detection, since it resulted more efficient than PCR CF1-CR2, at least in detecting *S. haematobium* DNA.

Thus, after DNA extraction using the Urine DNA Isolation Kit® all 73 patientś urine samples collected from immigrants were tested twice using both *S. mansoni* and *Schistosoma* spp. specific primers. In a first assay, PCR CF2-CR2 detected DNA of *S. haematobium* in eight samples (8/73; 10.9%) and PCR SmF-SmR only detected one *S. mansoni* positive sample (1/18; 5.5%). Unexpectedly, when we attempted a second assay on the same samples we obtained two positive results using PCR CF2-CR2, including two parasitologically confirmed *S. mansoni*-*S. haematobium* co-infections (2/73; 2.7%) and two specific *S. mansoni* PCR products were obtained when PCR SmF-SmR was applied (2/18; 11.1%). In conclusion, using the combination of the results with these two pairs of primers we found 13.7% and 16.7% sensitivity when urine samples from patients were analyzed with PCR CF2-CR2 and PCR SmF-SmR, respectively. We did not find any more positive results when samples were analyzed repeatedly using other PCR conditions (TD-PCR, etc) or when other previously successfully reported PCR for *Schistosoma* spp. detection in urine samples were tested [25].

Our results were much more scarce than expected and were found to be not reproducible with respect to either of the PCR protocols carried out on the same DNA samples. It seems logical to consider that the lack of positive results in our study cannot be due to the ineffectiveness of the PCR method for DNA amplification assays, since it has been previously reported as highly sensitive and specific not only in stool samples for *S. mansoni* detection [24], [43], but also in human urine samples for *Schistosoma* spp. detection [14]. We consider that our lack of positive results was probably due to the long-term frozen storage conditions of patientś urine samples. In this way, previous studies have shown that both temperature and storage time applied to urine samples affects the performance of DNA extraction and, therefore, a decrease in sensitivity of the PCR applied. Thus, Deelman *et al.* [44] found that the sensitivity of the PCR assay for amplification of the ACE gene in urine samples preserved at −20°C decreased considerably depending on the time of storage: 1 month, 18 months and 3 years. DNA stored for 1 month at −20°C performed equally well as fresh urine. However, DNA extracted from urine stored for 18 months at −20°C performed less well in the PCR assay. No DNA could be extracted from urine stored for 3 years at −20°C. In addition, the storage of urine at 4°C and 20°C for 24 and 48 hours did not result in successful amplification, indicating degradation of genomic DNA and, possibly, the extensive precipitation of urine components that interfere with the extraction procedure. The fact that our urine samples have been stored frozen much longer (ranging from 18 months to 7 years) from collection to analysis may have decisively contributed to the lack of positive results by PCR amplification. The positive results obtained with artificial urine samples, which were prepared *ad hoc* without any storage process at 4°C or freezing, seems to indicate that the effectiveness of the extraction of DNA is higher when it does not carry out these previous storage processes.

We conclude that long-term frozen human urine samples are probably not a good source for DNA extraction for use as a template in PCR detection of *Schistosoma* spp. regardless of the DNA extraction method used. Nevertheless, it could be possible that several other factors could lead to an apparent loss of PCR positivity, such as bacterial and/or fungal contamination during the storage of samples, or variation among aliquots in patientś sampling, or urine samples with a low egg count. Practical issues, such as the hydration status of the patients and the optimal time during the day for sample collection would need to be further evaluated. Our results should be considered for storing urine samples for a long time for subsequent molecular detection of *Schistosoma* spp. DNA.
